# A Network Pharmacology Study of the Multi-Targeting Profile of an Antiarrhythmic Chinese Medicine Xin Su Ning

**DOI:** 10.3389/fphar.2019.01138

**Published:** 2019-09-25

**Authors:** Taiyi Wang, Hamish Streeter, Xuan Wang, Ujang Purnama, Ming Lyu, Carolyn Carr, Yu-ling Ma

**Affiliations:** ^1^Oxford Chinese Medicine Research Centre, University of Oxford, Oxford, United Kingdom; ^2^Department of Physiology, Anatomy and Genetics, University of Oxford, Oxford, United Kingdom; ^3^Institute of Chinese Materia Medica, China Academy of Chinese Medical Sciences, Beijing, China

**Keywords:** Xin Su Ning, network pharmacology, phlegm-heat heart-disturbance, weight coefficient, cardiac arrhythmia, electrophysiology

## Abstract

Xin Su Ning (XSN) is a China patented and certified traditional Chinese herbal medicine used to treat premature ventricular contractions (PVCs) since 2005. XSN is formulated with 11 herbs, designed to treat arrhythmia with phlegm-heat heart-disturbed syndrome (PHHD) according to Chinese medicine theory. The rational compatibility of the 11 herbs decides the therapeutic outcome of XSN. Due to the multicomponent nature of traditional Chinese medicine, it is difficult to use conventional pharmacology to interpret the therapeutic mechanism of XSN in terms of clear-cut drug molecule and target interactions. Network pharmacology/systematic pharmacology usually consider all the components in a formula with the same weight; therefore, the proportion of the weight of the components has been ignored. In the present study, we introduced a novel coefficient to mimic the relative amount of all the components in relation with the weight of the corresponding herb in the formula. The coefficient is also used to weigh the pharmacological effect of XSN on all relative biological pathways. We also used the cellular electrophysiological data generated in our lab, such as the effect of liensinine and isoliquiritigenin on Na_V_1.5 channels; we therefore set sodium channel as one of the targets of these two components, which would support the clinical efficacy of XSN in treating tachyarrhythmia. Combining the collected data and our discovery, a panoramagram of the pharmacological mechanism of XSN was established. Pathway enrichment and analysis showed that XSN treated PHHD arrhythmia through multiple ion channels regulation, protecting the heart from I/R injury, inhibiting the apoptosis of cardiomyocyte, and improving glucose and lipid metabolism.

## Introduction

Arrhythmia is a disease featuring the abnormalities of frequency or rhythm of heart excitement caused by abnormal cardiac electrophysiological activities generated by the electrical conduction system of the heart. Symptoms of arrhythmia often include dizziness, breathlessness, and palpitations (Barsky, 2001). The presence of cardiac arrhythmias may at times suggest a specific underlying heart disease or even noncardiac pathological changes. The most common and important cause of cardiac arrhythmias is coronary artery disease (Atarashi and Hayakawa, 1996). In recent decades, even though plenty of non-chemical therapies have been developed, anti-arrhythmic drug is still the most common treatment. However, the proarrhythmic effect of the anti-arrhythmic drugs has been causing concerns on the safety of the arrhythmic patients.

As a complementary and alternative medicine, Chinese medicine plays an increasing role in the treatment of arrhythmias (Dong et al., 2017). Many proprietary Chinese medicines, such as Xin Su Ning capsule (Yuan and Zhou, 2000; Wang and Lu, 2008; Lin et al., 2011; Li and Zhang, 2015; Zhai et al., 2017), Wenxin Keli (Kalifa and Avula, 2012; Li and Guihua, 2018), and Shensong Yangxin capsule (Wang et al., 2011; Liu et al., 2014), have showed clear clinical antiarrhythmic efficacy comparable with the single chemical compound anti-arrhythmic drugs. However, the Chinese-patented antiarrhythmic medicines have rare adverse reactions being recorded.

Based on the theories of traditional Chinese medicine (TCM), a disease can be categorized from four fundamental dimensions, which is consisted of four pairs of relative concepts including yin–yang, exterior–interior, excess–deficiency, and cold–heat (Lu et al., 2004; Jiang et al., 2012). In terms of excess–deficiency categorization, arrhythmia can be divided into two main types of syndromes: excess syndrome and deficient syndrome (Chen and Ba, 2010). Deficiency syndrome is a TCM condition with weakness and lack of energy. Up to present, most of the marketed anti-arrhythmic Chinese medicines have been used to treat deficient syndrome, such as Wenxin Keli (Wang et al., 2016) and Shensong Yangxin capsule (Gu et al., 2005) for the deficiency of Qi and Yin, Xinbao Wan for the deficiency of Yang (Chen and Wu, 1996; Dong et al., 2005), and Tianwang Buxin Wan for the deficiency of Yin (Ni et al., 2019). Excess syndrome is a typical TCM syndrome caused by the accumulation of pathological phlegm and dampness in the body. Cardiac arrhythmia with typical clinical manifestation of phlegm-heat heart-disturbance (PHHD) syndrome takes a large proportion of the clinical treatment, and to the best of our knowledge, XSN is the only medicine for treating PHHD arrhythmia (Yuan and Zhou, 2000).

Xin Su Ning (XSN) is a multi-herbal medicine patented and launched in China since 2005 for treating cardiac ventricular arrhythmia, especially arrhythmias induced by cardiac ischemia and viral myocarditis (Zhai et al., 2017). XSN is comprised of 11 herbs: *Coptidis Rhizoma* (Huanglian, *Coptis chinensis* Franch.), *Pinelliae Rhizoma* (Banxia, *Pinellia ternata* [Thunb.] Makino), Poria (Fuling, *Poria cocos* [Schw.] Wolf), *Aurantii Fructus Immaturus* (Zhishi, *Citrus aurantium* L.), *Dichroae Radix* (Changshan, *Dichroa febrifuga* Lour.), *Nelumbinis Plumula* (Lianzixin, *Nelumbo nucifera* Gaertn.), *Sophorae flavescentis Radix* (Kushen, *Sophora flavescens* Ait.), *Artemisiae annuae Herba* (Qinghao, *Artemisia annua* L.), *Ginseng Radix et Rhizoma* (Renshen, *Panax ginseng* C. A. Mey.), *Ophiopogonis Radix* (Maidong, *Ophiopogon japonicus* (L. f) Ker Gawl.), and *Nardostachyos Radix et Rhizoma* (Gancao, *Glycyrrhiza uralensis* Fisch.).

However, the complexity of the chemical composition of multi-herbal TCM formula brings great difficulties to the pharmacological research. Network pharmacology has been one of the approaches to reveal the complex pharmacological mechanisms behind the multitargeting properties by the multicomponent medicines (Li et al., 2011). TCM formulas are well known by the characteristics of multi-herbal/component, which would exert its clinical efficacy through multiple targeting, hence multi-pharmacological actions (Leung et al., 2014). However, most of the studies were centered on attributing the edge between component and target and considering the weight values of all the components as the same (Shao and Zhang, 2013). A good TCM formula would be formed with the right herbs in an accurate proportion that would produce the best possible clinical efficacy with none or minimal toxicity. Therefore, each herb in a formula would be indispensable in evaluating the clinical efficacy of the formula as well as in a network pharmacological mechanism mapping. Furthermore, in terms of compound formula which consists of multiple herbs, the target spectrum (target distribution of all monomer components) determines the therapeutic range of the formula, and high-weight components may determine the therapeutic direction.

In this study, we tried to introduce a parameter, weight coefficient, to mimic the proportion of all the encompassed components and resort their effects on different targets and pathways. We used the data from multiple bioassay databases, combining with the results of pharmacological assays of high-weight coefficient components; a closer-to-the-fact pharmacological panorama was constructed.

## Materials and Methods

### XSN Chemical Component Library Building

All of the chemical monomer components in the 11 herbs of XSN were retrieved from the book Chemical Components of Source Plants in Traditional Chinese Medicine (Zhou et al., 2009) and the database: TCM Systems Pharmacology Database and Analysis Platform (TcmSP™, http://lsp.nwu.edu.cn/tcmsp.php) (Ru et al., 2014). In addition, four important pharmacology-related properties of the collected components were also obtained from admetSAR (Cheng et al., 2012; Yang et al., 2018), including MW, ALogP, Hbond donor count, and Hbond acceptor count, Rotation bond count, and human oral bioavailability (HOB). The proportion of each herb in XSN formula was retrieved from the patent of XSN (Wang and Mao, 2018), and the contents of the main active components in all the 11 herbs were retrieved from published papers, and all the other components without quantitative data were set to be equal to the lowest quantity value of known components (details see **Supplement Table 1**).

### Calculation of Weight Coefficients

Component weight coefficient:

$$w_{i,j}=\frac{m_{i}}{\Sigma_{i}^{n}m_{i}}\times \frac{C_{i,j}}{M_{j}}\times p_{j}(OB)\times 10^{9}$$

*w*_i,j_ is the weight coefficient of component *j* in herb *i*, and *m_i_* is the weight of herb *i* in XSN formula, *n* is the total count of herbs in XSN, *C_i,j_* is the content of component *j* in herb *i*, and *M_j_* is the molecular weight of component *j*, and *p_j_*(*OB*) is the predicted probability of positive human OB of component *j*.

The weight coefficient is the product of three elements: $\frac{m_{i}}{\sum_{i}^{n}m_{i}}$ is the proportion of each herb in the XSN formula, $\frac{c_{i,j}}{M_{j}}$ represented the molar concentration of each component in 1 gram of raw herb in XSN formula, and *pj(OB)* is the probability of the bioavailability of each component reaching 30% of the total oral administration. Therefore, the coefficient *w_i,j_* is a relative quantity among all the components of the 11 herbs in XSN formula to reflect the proportional relationships of the components existed in the body.

Since the activity of the multiple herbal medicine involved in multiple targeting, and each target was usually bound by multiple components. Weight coefficient of one single target in this paper was represented by the sum of all the weight coefficients of the components interacting with this target. Furthermore, weight coefficient of each pathway was also represented by the sum of all the weight coefficients of all targets in this pathway.

### Establishment of the Relationships Between Component-Target and Zheng Target

All the relative targets of each component in the 11 herbs of XSN were retrieved from BindingDB database (https://www.bindingdb.org/) as the component-target relationship library of XSN (Gilson et al., 2016). Since Zheng (TCM syndrome) is represented by the series of characteristics with clinical manifestations and symptoms (hereinafter referred to as TCM symptom), all the 10 TCM symptoms of PHHD arrhythmia referred in the indications of XSN capsule and the standard names of their corresponding modern medicine symptoms (MM symptom) were collected from SymMap database (https://www.symmap.org/) (Wu et al., 2018). All the target genes relative to each MM symptom above were obtained from the Human Phenotype Ontology Database (https://hpo.jax.org/app/) as the target-Zheng relationship library of PHHD arrhythmia.

### Network Construction and Analysis

Two networks were constructed: the networks of PHHD target and XSN component targets. All the networks were visualized by Cytoscape 3.7.0 (http://www.cytoscape.org), which is an open-source project for complex network construction and visualizing and analyzing (Shannon et al., 2003). Pathway enrichment was carried out using Reactome pathway database (https://www.reactome.org) (Fabregat et al., 2017; Fabregat et al., 2018); the pathways with FDR < 0.05 were kept in the result.

### Chemicals and Solutions

All the chemical agents were purchased from Sigma–Aldrich, and all high-weight coefficient components were obtained from Chengdu Herbpurify Co., Ltd. The intracellular buffer contained (in mM): KCl 120, MgCl_2_ 2, CaCl_2_ 1, Na_2_ATP 3, EGTA 11, HEPES 10, and pH 7.2 corrected with 5M NaOH. The extracellular buffer contained (in mM): NaCl 112, NaH_2_PO_4_•H_2_O 1, KCl 5.4, HEPES 5, NaHCO_3_ 24, glucose 10, MgCl_2_ 1.2, CaCl_2_ 1.8, and pH 7.4 corrected with 5M NaOH. Components experimented in this paper were dissolved in the external buffer, and for the dose–response research, the concentrations of the compounds studied were ranging from 1 to 100 μM in the external buffer solution.

### Electrophysiological Research

CHL cells stably expressing the α-subunit of human Na_V_1.5 (SCN5A) were used for electrophysiological assays. Patch clamp assays were carried out at room temperature (∼ 22 to 24°C), and the cells were superfused with the extracellular buffer at a rate of 2 ml/min. Patch pipettes were pulled from borosilicate glass (Harvard Apparatus, UK) using a DMZ-Universal Puller (Zeitz-Instruments, Germany); the average pipette resistance was 3–5 MΩ. The control currents were recorded 5 minutes after the whole-cell configuration was achieved using an Axopatch™ 200B Amplifier (Molecular Devices, USA).

### Data Analysis

Data was analyzed and illustrated using pCLAMP 10.3 software (Axon Instruments, Inc.) and Origin 9.1. For each protocol and condition, at least five cells were tested. Data values are presented as mean ± standard error of the mean (SEM). The difference between the control and the effect of a compound was statistically tested using Student’s unpaired t-test. *P* < 0.05 was deemed to be statistically significant. Where results are not statistically significant, the actual *p*-value is provided.

## Results

### XSN Component Library

**Figure 1:**
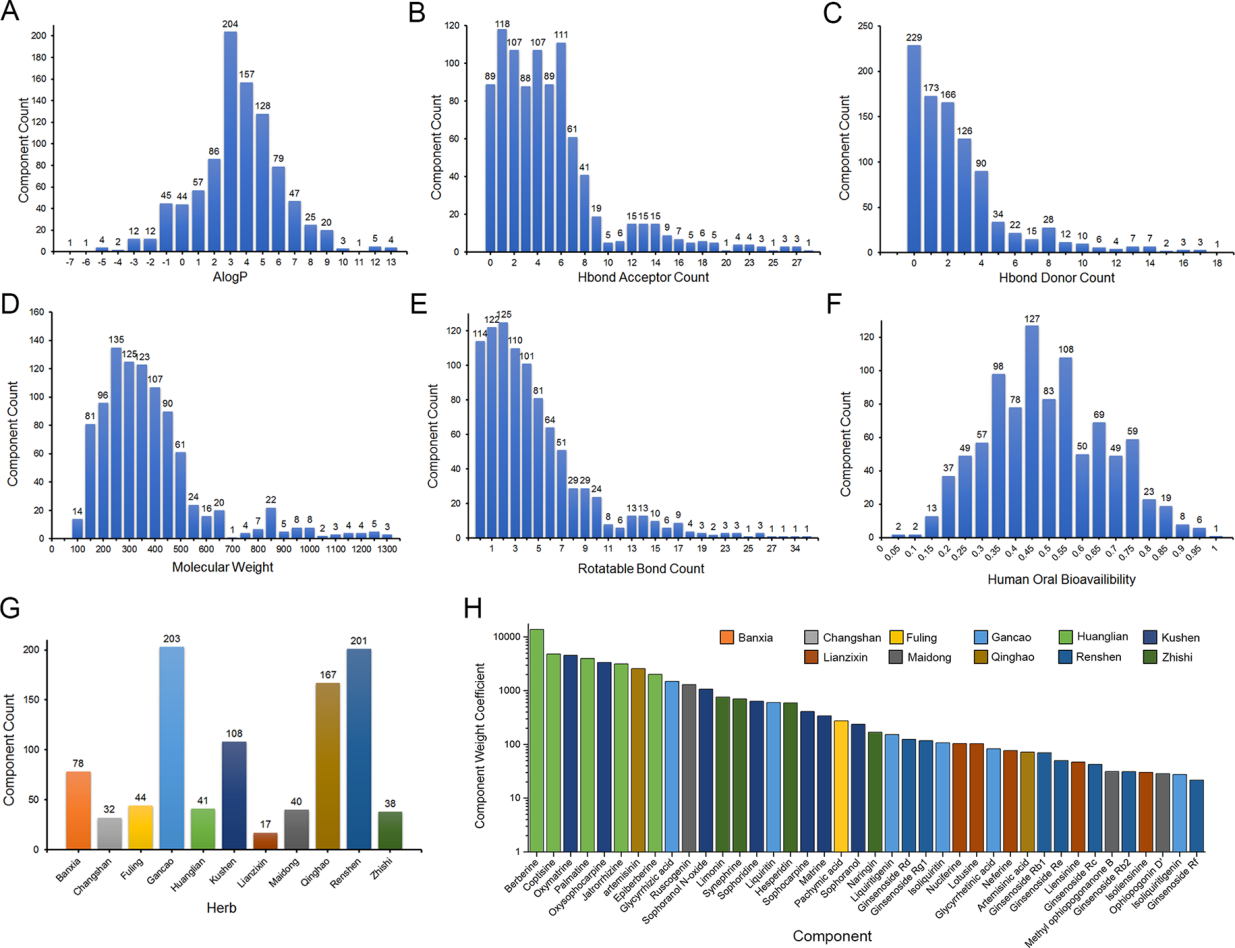
Chemical properties statistics of components in the 11 herbs of XSN. **(A)** AlogP, **(B)** Hbond donor count, **(C)** Hbond acceptor count, **(D)** Molecular weight (MW), **(E)** human oral bioavailability, **(F)** rotatable bond count, **(G)** component count of each herb collected in XSN component library, and **(H)** weight coefficient of top 40 weight components in 11 herbs. The different colors means the source herb of components, which is same as **(G)**.

Nine hundred sixty-three monomer components from 11 herbs of XSN were collected from Chemical Components of Source Plants in Traditional Chinese Medicine and TCMSP database (**Supplement Table 1**). Chemical properties including AlogP, Hbond donor and acceptor count, molecular weight (MW), human OB, and rotatable bond count were calculated by AdmetSar 2.0 as described above, analyzed and shown as **Figures 1A–F** (**Supplement Table 1**). The component accounts of each herb were analyzed and shown in **Figure 1G**. The content of monomer components was obtained from published chemical constituent research indexed by PubMed (https://www.ncbi.nlm.nih.gov/pubmed) and CNKI (http://www.cnki.net/) (**Supplement Table 1**). The contents of 47 of 963 components above were quantified in the reports, and the content values were abstracted from the references. The contents of the remaining 916 components were set as the lowest value of the content reported components. The most abundant component berberine was 2.82×10^6^ times that of neohesperidin which was with the lowest content in the 47 reported components. The weight coefficients for all the components were calculated, and top 40 with references were listed and shown in **Figure 1H** with higher weight components.

### XSN Target Spectrum

**Figure 2:**
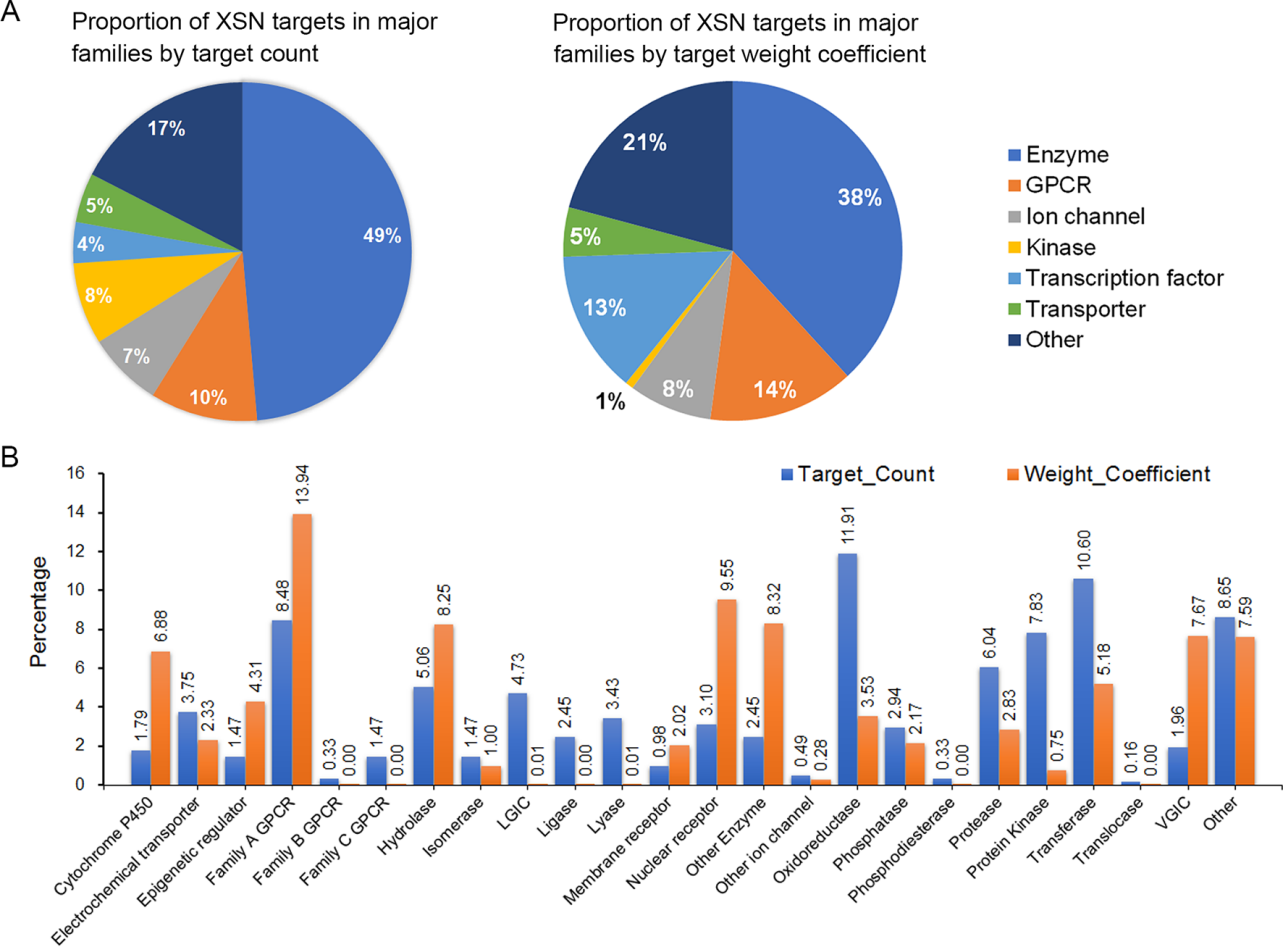
The targeting spectrum of XSN. **(A)** Statistic and major classifications of XSN target spectrum by target count and **(B)** statistic and secondary classifications of XSN target spectrum. The X axis of **(B)** is labeled with targets including family A GPCR, rhodopsin-like GPCRs; family B GPCR, secretin-like GPCRs; family C GPCR, metabotropic glutamate receptor family; LGIC, ligand-gated ion channel; VGIC, voltage-gated ion channel.

Two thousand eight hundred thirty-five component-target relationship data were obtained, covering 487 monomer components and 618 targets (**Supplement Table 2**). The classification of targets was analyzed using the ChEMBL hierarchical target classification system (Bento et al., 2014), and the classification of enzymes were updated according to the ENZYME database (Bairoch et al., 2004). Major and secondary classifications of target spectrum were shown as **Figure 2** (**Supplement Table 3**). The proportion of targets were grouped by the means of target count in **Figure 2**, which shows the proportion of different target categories and also shows the differences between statistics by target count and weight coefficient, especially for cytochrome P450, hydrolase, nuclear receptor, enzymes, oxidoreductase, protease, protein kinase, and VGIC (proportion > 5% and fold change >3).

### PHHD-Arrhythmia Target Spectrum

Since Zheng (TCM syndrome) is represented by the series of characteristics with clinical manifestations and symptoms (hereinafter referred to as TCM symptom); all the 10 TCM symptoms of PHHD arrhythmia and their relative modern medicine symptoms (MM symptom) were collected: Xin Ji (palpitations), Xiong Men (respiratory distress), Xin Fan (boredom), Yi Jing (panic attack), Kou Gan (xerostomia), Kou Ku (bitter taste in the mouth), Shi Mian (insomnia), Duo Meng (dreaminess), Xuan Yun (vertigo), and Mai Jie Dai (knotted or regularly intermittent pulse) (**Table 1**, **Supplement Table 4**).

**Table 1:** The relative relationship between TCM symptoms and modern medicine symptom.

TCM symptom ID	TCM symptoms	MM symptom ID	MM symptoms	UMLS ID	Synonyms	UMLS ID	HPO ID	Target number
SMTS01333	*Xin Ji*	SMMS00282	Palpitations	C0030252			HP:0001962	52
SMTS01384	*Xiong Men*	SMMS00863	Chest Heaviness	C0742339	Respiratory distress	C0013404	HP:0002098	108
SMTS01324	*Xin Fan*	SMMS00381	Boredom	C0006019				
SMTS01567	*Yi Jing*	SMMS00119	Panic Attack	C0086769			HP:0025269	17
SMTS00573	*Kou Gan*	SMMS00144	Xerostomia	C0043352			HP:0000217	42
SMTS00580	*Kou Ku*	SMMS00585	Halitosis	C0018520	Abnormality of taste sensation	C4025879	HP:0000223	6
SMTS00970	*Shi Mian*	SMMS00033	Insomnia	C0917801			HP:0100785	12
SMTS00211	*Duo Meng*	SMMS00738	Nightmares	C0028084				
SMTS01439	*Xuan Yun*	SMMS00115	Vertigo	C0042571			HP:0002321	72
N/A	*Mai Jie Dai*				Irregular heart beat		0011675	317

TCM, traditional Chinese medicine; MM, modern medicine; UMLS, Unified Medical Language System; HPO, human phenotype ontology.

TCM symptom Kou Ku means bitter taste in the mouth, but not bad breath in the mouth; therefore, the MM symptom halitosis retrieved from SymMap was replaced by another keyword: abnormality of taste sensation. Mai Jie Dai included two types of pulses: Jie Mai is knotted or bound pulse, which is slow, relaxed and stops at irregular intervals. Jie Mai represents an irregular beat or palpitation stemming from the heart. Dai Mai means the pulse is with regularly intermittent abnormality, which also suggests the patients with this pulse have advanced heart disease according to modern medicine. Therefore, an alternative keyword irregular heartbeat was used to represent the TCM symptom Mai Jie Dai.

**Figure 3:**
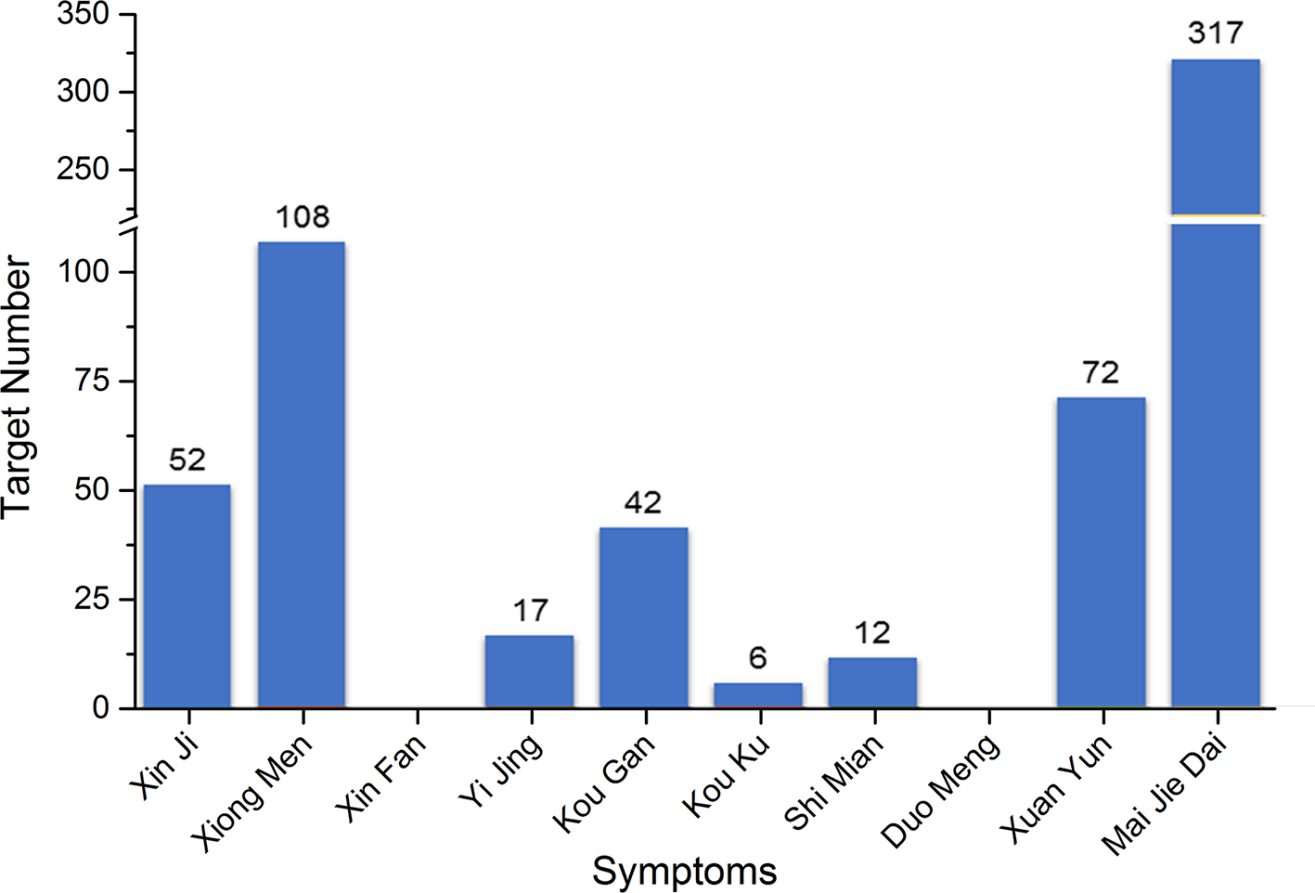
Statistics of target count for each TCM symptom of PHHD arrhythmia.

Five hundred two targets in total for PHHD arrhythmia were collected as PHHD-arrhythmia target spectrum; statistics of target count for each TCM symptom were shown in **Figure 3** (**Supplement Table 4**).

To obtain the pharmacological network of XSN on treating PHHD arrhythmia, 40 targets were obtained in the common set of XSN target spectrum and PHHD-arrhythmia target spectrum (**Table 2**). All the components interact with the 40 targets above consist of the panoramagram of the pharmacological mechanism of XSN for treating PHHD arrhythmia.

**Table 2:** The common genes between XSN target spectrum and PHHD-arrhythmia target spectrum.

Gene_Symbol	TCM_Symptom	Target_Weight_Coefficient	UniProt_ID	ChEMBL_ID	Target_Name	Classification_1	Classification_2
CALM1	Maijiedai, Xuanyun	790.2976034	P0DP23	CHEMBL6093	Calmodulin	Other	Unclassified protein
KCNH2	Maijiedai, Xinji	13942.14102	Q12809	CHEMBL240	HERG	Ion channel	VGIC
TP53	Yijing, Maijiedai, Xinji	13900.81344	P04637	CHEMBL4096	Cellular tumor antigen p53	Transcription factor	Other
ABCC8	Maijiedai	13817.52659	Q09428	CHEMBL2071	Sulfonylurea receptor 1	Transporter	Other
KCNJ11	Maijiedai	13817.40799	Q14654	CHEMBL1886	Potassium channel, inwardly rectifying, subfamily J, member 11	Ion channel	VGIC
KCNJ5	Maijiedai	169.079052	P48544	CHEMBL3038488	Kir3.1/Kir3.4	Ion channel	VGIC
CACNA1B	Maijiedai	593.6573686	Q00975	CHEMBL4478	Voltage-gated N-type calcium channel alpha-1B subunit	Ion channel	VGIC
TARDBP	Kougan	2584.201263	Q13148	CHEMBL2362981	TAR DNA-binding protein 43	Other	Unclassified protein
JAK2	Xuanyun	2032.705321	O60674	CHEMBL2971	Tyrosine-protein kinase JAK2	Kinase	Protein Kinase
TNNC1	Maijiedai	2017.925664	P63316	CHEMBL2095202	Troponin, cardiac muscle	Other	Other
TNNI3	Maijiedai	2017.925664	P19429	CHEMBL2095202	Troponin, cardiac muscle	Other	Other
TNNT2	Maijiedai	2017.925664	P45379	CHEMBL2095202	Troponin, cardiac muscle	Other	Other
GMNN	Xiongmen	95.88983101	O75496	CHEMBL1293278	Geminin	Other	Unclassified protein
SMAD3	Maijiedai	95.88983101	P84022	CHEMBL1293258	Mothers against decapentaplegic homolog 3	Other	Unclassified protein
PPARG	Maijiedai, Xinji	83.46168023	P37231	CHEMBL235	Peroxisome proliferator-activated receptor gamma	Transcription factor	Nuclear receptor
GJA1	Maijiedai	83.29977996	P17302		Gap junction alpha-1 protein	Other	Unclassified protein
PTPN11	Maijiedai	83.26574007	Q06124	CHEMBL3864	Protein-tyrosine phosphatase 2C	Enzyme	Phosphatase
LMNA	Maijiedai	83.14071435	P02545	CHEMBL1293235	Prelamin-A/C	Other	Unclassified protein
TSHR	Maijiedai	83.14071435	P16473	CHEMBL1963	Thyroid-stimulating hormone receptor	GPCR	Family A GPCR
ATXN2	Kougan	12.74911666	Q99700	CHEMBL1795085	Ataxin-2	Other	Unclassified protein
SOD1	Kougan	1.975815734	P00441	CHEMBL2354	Superoxide dismutase	Enzyme	Oxidoreductase
SDHA	Yijing, Maijiedai, Xinji, Xuanyun	1.990418899	P31040	CHEMBL5758	Succinate dehydrogenase (ubiquinone) flavoprotein subunit, mitochondrial	Enzyme	Oxidoreductase
PON1	Kougan	1.732079071	P27169	CHEMBL3167	Serum paraoxonase/arylesterase 1	Enzyme	Hydrolase
KYNU	Maijiedai	1.649920118	Q16719	CHEMBL5100	Kynureninase	Enzyme	Hydrolase
IFNG	Maijiedai	1.695300734	P01579	CHEMBL3286073	Interferon gamma	Other	Other
CHRM3	Kougan	0.769622544	P20309	CHEMBL245	Muscarinic acetylcholine receptor M3	GPCR	Family A GPCR
SCN5A	Maijiedai, Xinji	76.0137842	Q14524	CHEMBL1980	Sodium channel protein type V alpha subunit	Ion channel	VGIC
DAO	Kougan	1.396943488	P14920	CHEMBL5485	D-amino-acid oxidase	Enzyme	Oxidoreductase
CPT2	Maijiedai, Xiongmen	1.020829585	P23786	CHEMBL3238	Carnitine palmitoyltransferase 2	Enzyme	Transferase
CTNNB1	Xuanyun	1.020829585	P35222	CHEMBL5866	Catenin beta-1	Other	Unclassified protein
NAGS	Xiongmen	1.020829585	Q8N159		N-acetylglutamate synthase, mitochondrial	Enzyme	Transferase
TTR	Maijiedai	1.001902474	P02766	CHEMBL3194	Transthyretin	Other	Other
COL3A1	Xuanyun	0.45282272	P02461	CHEMBL2364188	Collagen	Other	Other
SLC1A3	Xuanyun	0.520835938	P43003	CHEMBL3085	Excitatory amino acid transporter 1	Transporter	Electrochemical transporter
HNF4A	Maijiedai	0.386540369	P41235	CHEMBL5398	Hepatocyte nuclear factor 4-alpha	Other	Unclassified protein
AKT1	Maijiedai	0.26386963	P31749	CHEMBL4282	Serine/threonine-protein kinase AKT	Kinase	Protein kinase
GAA	Maijiedai	0.261304876	P10253	CHEMBL2608	Lysosomal alpha-glucosidase	Enzyme	Hydrolase
LDLR	Maijiedai	0.132692707	P01130	CHEMBL3311	LDL receptor	Other	Membrane receptor
EGF	Xuanyun	0.159065607	P01133	CHEMBL5734	Pro-epidermal growth factor	Other	Unclassified protein

### Pharmacodynamic Research of High-Weight Components in XSN

Based on our previous study, XSN is a class III anti-arrhythmic drug supported by the prolongation of the action potential of cardiac myocytes through blocking hERG channel (Ma et al., 2016; Wang et al., 2017), and it also showed class I antiarrhythmic property of blocking sodium channels (Wang et al., 2019b). However, the available pharmacological research data of all the components in the 11 herbs of XSN reported and recorded in all the databases are not sufficient to correlate with the clinical antiarrhythmic efficacy and our discovery that inhibit human cardiac sodium current, Na_V_1.5 channel (encoded by gene SCN5A) with total target weight coefficient of 1.32 (Wang et al., 2019b). Comparing with the effect of hERG channel (encoded by KCNH2), total target weight coefficient of hERG is 14838.89; both of them are with similar IC_50_ values generated by XSN, which can be considered as a combination of all the components. Thus, we screen the set of high-weight components with reported content in corresponding herbs on inhibiting Na_V_1.5.

**Figure 4:**
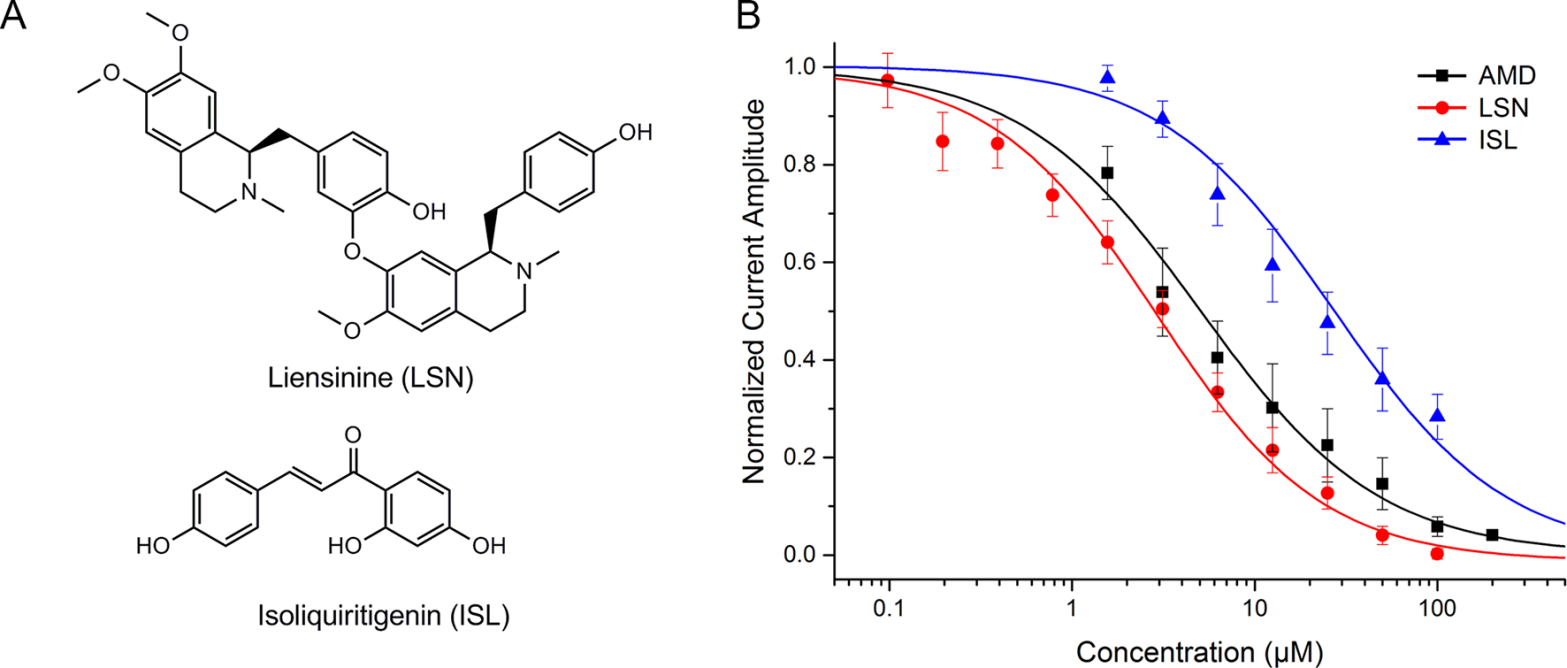
**(A)** Chemical structure of liensinine (LSN) and isoliquiritigenin (ISL). **(B)** Dose–response curve of the inhibition on human Na_V_1.5 channel by amiodarone (AMD, n=5), LSN (n=6), and ISL (n=8).

Two high-weight active components: liensinine (LSN, PubChem CID: 160644) and isoliquiritigenin (ISL, PubChem CID: 638278) were detected using electrophysiological approaches. LSN is the No. 35 high-weight component, which blocked human Na_V_1.5 channel dose-dependently with an IC_50_ of 3.58 ± 0.36 μM (**Figure 4**). ISL reduced peak I_Na_ concentration dependently, with a median inhibitory concentration (IC_50_) of 10.11 μM ± 1.12 μM. The positive control amiodarone (AMD) blocks Na_V_1.5 channel with IC_50_ = 5.05 ± 0.55 μM.

### Panoramagram of the Pharmacological Mechanism of XSN

**Figure 5:**
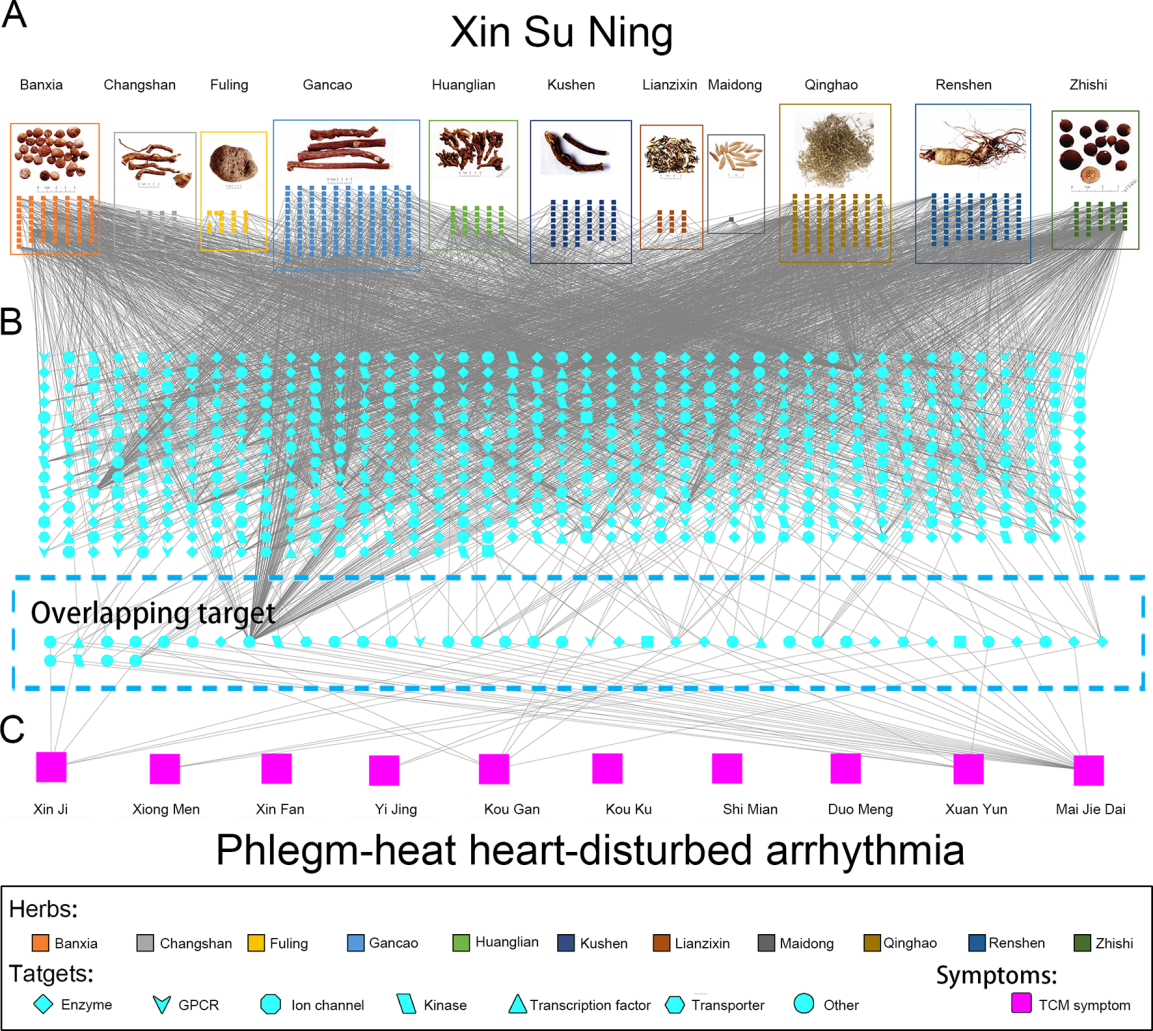
Panoramagram of the pharmacological mechanism of XSN for treating PHHD arrhythmia. This panoramagram illustrated the connections among the XSN formula, herbs, components, targets, symptoms, and PHHD arrhythmia. **(A)** XSN formula and components. All the 11 herbs were shown as figures in the box with different colors, and the components of each herb were shown as small square with the same color as their source herb. **(B)** XSN target spectrum. All the targets were corresponding to the component in panel A; the 41 targets showed in the dash line box in the bottom is the overlapping target set between XSN target spectrum and PHHD-arrhythmia target spectrum. Targets were shown in different shapes according to the classifications as indicated in the keys. **(C)** Arrhythmia with PHHD Zheng and relative TCM symptoms. Ten TCM symptoms of PHHD arrhythmia were represented by large pink squares in the bottom panel.

Including the pharmacological effects of LSN and ISL, a panoramagram of the integrative pharmacological mechanism of XSN was interpreted (shown as **Figure 5**). The pharmacological network consisted of 963 components, 618 targets, and 10 symptoms. This panoramagram illustrated the network pharmacological relationships among the XSN formula, herbs, components, targets, symptoms, and PHHD arrhythmia.

### Pathways Involved in the Pharmacological Mechanism of XSN on Treating PHHD Arrhythmia

**Figure 6:**
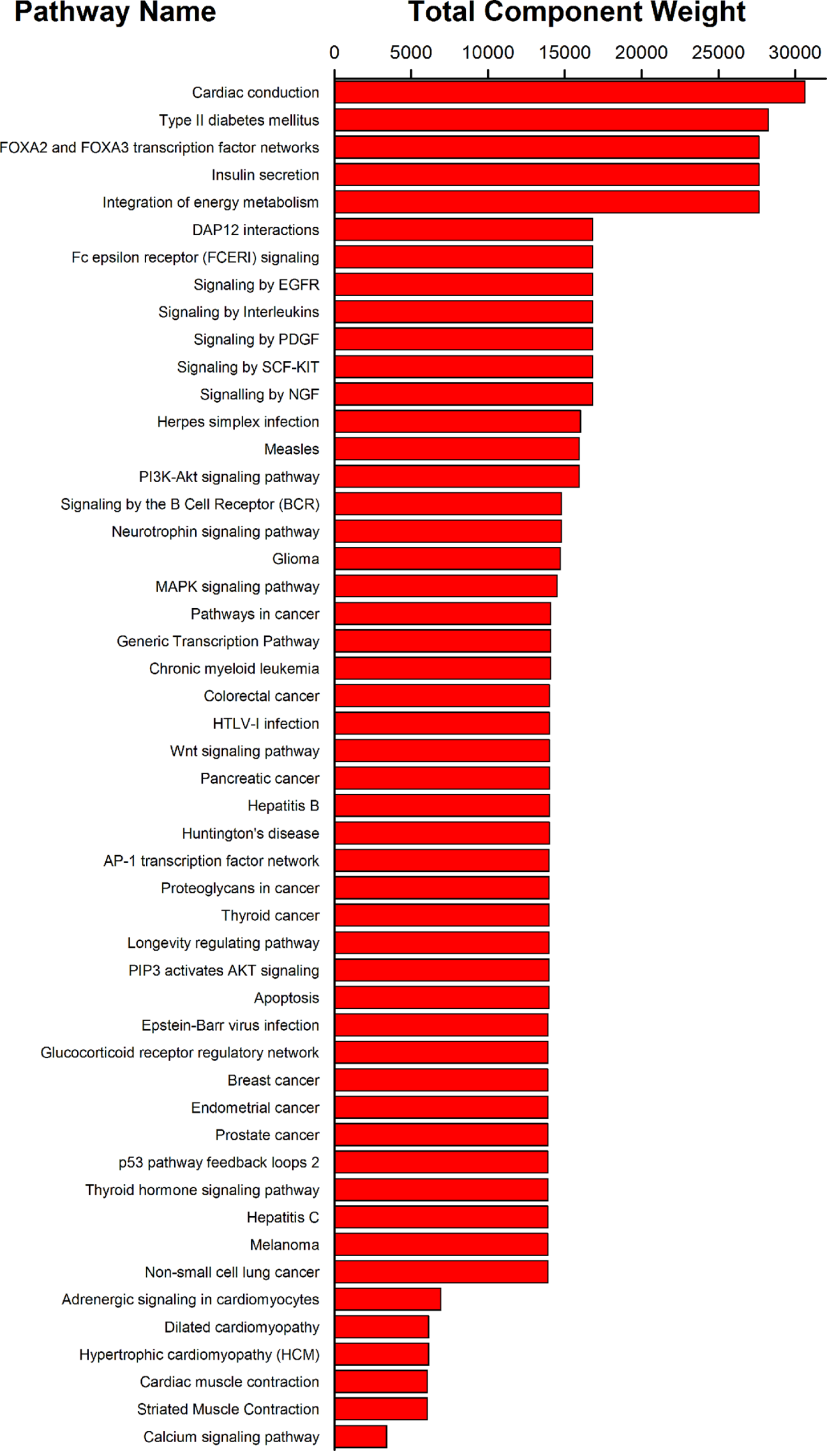
Pathway enrichment results of 40 targets of XSN on treating PHHD arrhythmia. Top 50 pathways were shown here; and all were sorted by pathway weight coefficient.

All the 40 common targets between XSN target spectrum and PHHD-arrhythmia target spectrum were selected to carry out pathway enrichment with Reactome application in Cytoscape. 117 pathways with FDR less than 0.05 were obtained and resort by total weight coefficients (**Figure 6**, **Supplement Table 5**).

**Figure 7:**
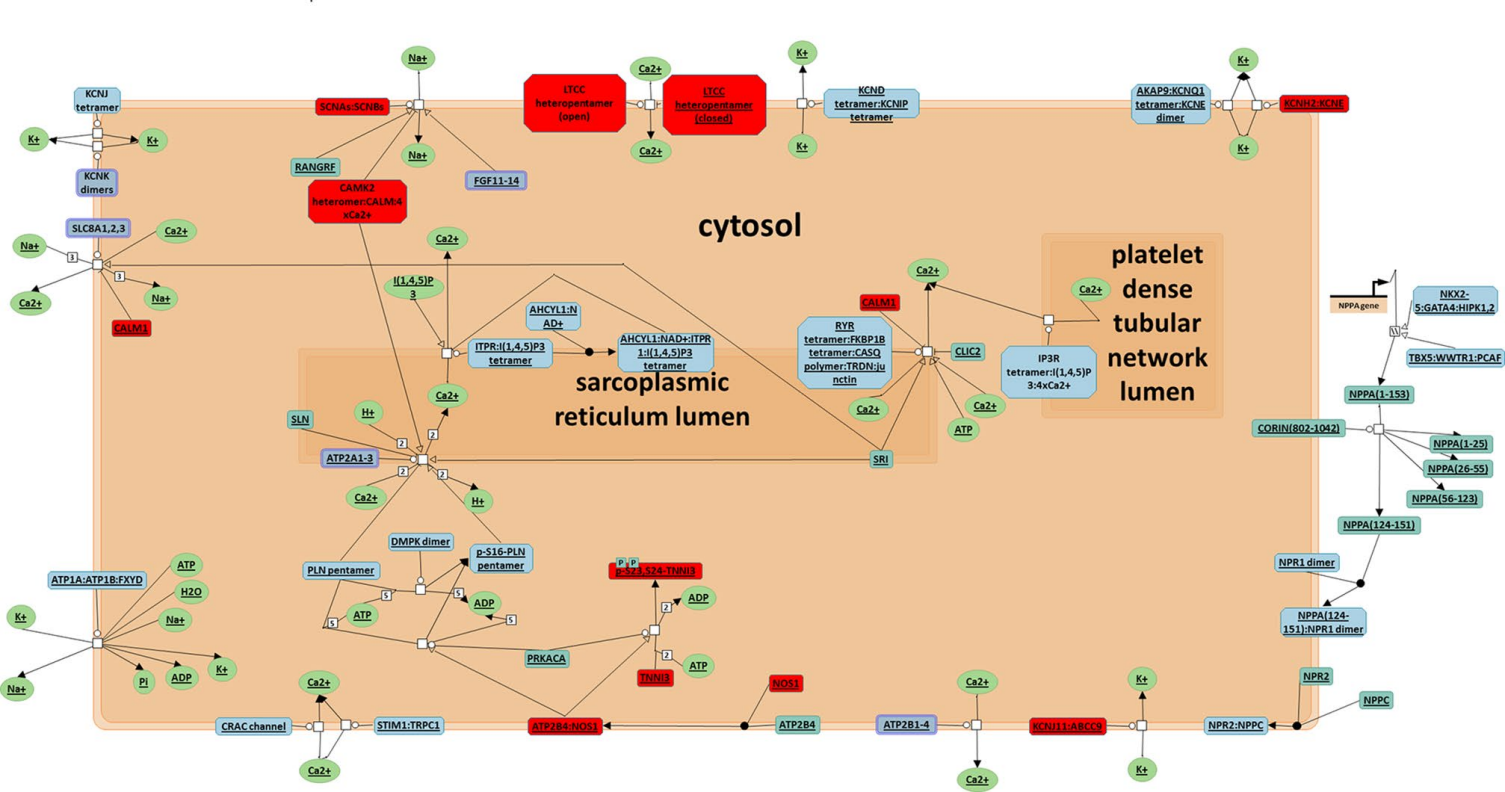
Cardiac conduction pathway. Top 1 pathway in the rank of total target weight coefficient, all the target regulated in this pathway were marked in red (Jassal, 2014).

To better understand the antiarrhythmic therapeutic efficacy of XSN, cardiac conduction pathway is calculated to hold the highest total weight coefficient in the list of resorting pathway total weight coefficient. It was shown in **Figure 7** and all the components in XSN involved in regulating this pathway were marked in the figure.

## Discussion

Tan-Re-Rao-Xin Zheng is named as PHHD syndrome in English. Based on the traditional Chinese Medicine theories, PHHD is defined as that heat having existed in the body for a period of time without distributing leads to the body's fluids being concentrated as Phlegm. The concept *Heart* in the Chinese medicine mainly refers to the circulatory system and nervous system, and they are easily to be blocked or obstructed to cause malfunction (Jiao et al., 2015). This is why PHHD is classified as an “excess” syndrome; the heat and the phlegm must be cleared to treat the symptoms in circulatory system and neuronal system successfully.

Physical symptoms of PHHD are usually consist of the followings (Yongdun et al., 2005; Ren et al., 2012; Xing et al., 2015): (1) insomnia, difficulty in falling asleep and frequent dream-disturbed waking. (2) Palpitations, the heart rhythm becomes out of control, e.g., PVCs or tachycardia. However, it depends on the severity of how much the heart had been disturbed by the phlegm fire; the clinical manifestation could range from incidental PVC to frequent PVC or from normal heart rate to tachycardia. In the region of the heart, there is often a sensation of heat or discomfort and oppressed chest, feeling difficult to breath. (3) Tongue, tongue body is red, especially at the tip, which is often swollen and painful. The tongue coating is yellow and thick. There is very often a mid-line crack extending to the tip, with yellow moss on it. Hypertension—high blood pressure—is often a feature of heart phlegm fire. (4) Pulse, slippery, and rapid and overflowing which means “excess”; it often appears after the first stage of infectious disease and shows excess heat. Complexion is red; eyes are bloodshot. Some mental symptoms could also happen, like boredom or even hypomania which also depends on the severity of phlegm-heat disturbing the neural system (which is also part of the *Heart* in TCM theory). Therefore, arrhythmia with obvious clinical signs of PHHD is a particular condition that the patient suffering from phlegm-heat, and the circulation system would be mostly influenced.

XSN capsule originated from the famous compound formula Huanglian Wendan (HLWD) Decoction recorded in 150 years ago, which has the effect of removing phlegm, blood stasis, and fire toxin. Huanglian, Banxia, Fuling, Gancao, and Zhishi are from the original formula HLWD decoction; Changshan, Lianzixin, Qinghao, and Kushen were added to make XSN formula more inclined to the circulation system. Therefore, XSN was not only a combination of anti-arrhythmic monomer components, but also a complex therapeutic system to regulate biological/pathological process relative to PHHD syndrome.

The compatibility principle of the herbs in XSN also reflects another key property of Chinese herbal medicine: amount/proportion of herbs in compound formula. For a better understanding of the pharmacological mechanism of XSN, we introduced a novel parameter, *weight coefficient*, to mimic the compatible combination of all the chemical components in the 11 herbs in XSN. As mentioned in methods section, weight coefficient is the product of proportion of herbs in the compound formula, content of each component in relative herb and the predicted probability of human OB of each component. Weight coefficient is not the absolute concentration of each chemical component but a relative content among them, which means the ranked order of weight coefficient of each component can be considered as the extent how much a component involved in the pharmacological mechanism of XSN. The weight coefficient of each component reflects its molar concentration in the blood; therefore, the higher the weight coefficient, the more molecules will bind to the targets. However, the binding affinity of the component to the target remains to be tested pharmacologically.

In this study, we used network pharmacological approach to visualize the complex pharmacological mechanism of XSN on treating PHHD arrhythmia. All the data were collected from databases and books to assure the quality and reliability of all the relationships in the pharmacological network. Based on our previous study, XSN can be classified as class III antiarrhythmic drug, and with some pharmacological property of class I drug (Sweeney et al., 2019; Wang et al., 2019a; Wang et al., 2019b). The application of XSN resulted in significant morphological changes of the ECG trace of isolated rat heart featured by the suppression of the R wave amplitude. The QRS complex represents ventricular depolarization as a result of activation of Na_V_1.5. Our previous study attributed the decrease in R wave amplitude to XSN dependent inhibition of I_Na_; when whole-cell sodium currents were elicited using a depolarizing pulse protocol, it was evident that XSN inhibited I_Na_ concentration dependently and reversibly. However, the data from reported research recorded in the databases were not sufficient to support all the antiarrhythmic effect of XSN; we, therefore, carried out a small scale component screening on the target of class I antiarrhythmic drug, human Na_V_1.5 channel. Given previous collected in this paper did not provide enough weight coefficient on Na_V_1.5 channel, some unidentified high-content components should target Na_V_1.5. Thus, we screened a series of high-content components listed in **Figure 1H**, to replenish the component-target information. LSN and ISL were identified as active components on Na_V_1.5 channel in the present study. Briefly speaking, the pharmacologically defined inhibition of Na_V_1.5 by the active components of the 11 herbs in XSN suggests that Lien and ISL bind to the channel in its inactivated state which reduces the I_Na_ amplitude. The total weight coefficient of Na_V_1.5 is 76.013, which is still much lower than hERG 13942.141. The reason is understandable that, based on the content of components in the mixture of raw herbs of XSN, the weight of Huanglian (334g/2,377g mixture of raw herbs) is much more than Lianzixin (42g/2,377g mixture of raw herbs) and Gancao (167g/2,377g mixture of raw herbs); meanwhile, the concentration of berberine is much higher than LSN and ISL in relative herbs (details see **Supplement Table 1**). Secondly, the experimental inhibitory effect of Na_V_1.5 by XSN was carried on directly on isolated heart or cell lines without influenced by absorption, distribution, metabolism, and excretion; the predicted oral bioavailability also lower down the weight coefficients of LSN and ISL. Technically, more active components inhibiting Na_V_1.5 were still undiscovered, and further research on XSN is still needed.

Including the pharmacological effects of LSN and ISL, we drew a panoramagram of the integrative pharmacological mechanism of XSN with 475 components targeting 617 targets. Among the 617 targets, 40 targets were relative to PHHD arrhythmia. To explain the mechanism, we carried out pathway enrichment instead of subnetwork analysis, 116 pathways were obtained, and top 50 were shown as **Figure 6**.

As an antiarrhythmic drug, XSN showed multiple therapeutic effects. From the standpoint of TCM treatment, both tip and root causes can be reflected in the pathways, which can also be considered as quick-acting and long-acting mechanisms. First, ion channels play essential role in terminating abnormal electrical activity in arrhythmic heart. Based on the results from the analysis above, the mechanism on cardiac electronic activity may involve such targets: TNNI3, CACNA1S, SCN5A, KCNH2, KCNJ11, and CALM1. Na_V_1.5, Ca_V_1.2, and hERG are three key ion currents in cardiac electric conduction during depolarization and repolarization. Troponins I (encoded by TNNI3) is integral to cardiac muscle contraction, which is also used as diagnostic and prognostic indicators in the management of myocardial infarction and acute coronary syndrome. Calmodulin 1 (encoded by CALM1) mediates the regulation of plenty of enzymes, ion channels, aquaporins, and other proteins acting with calcium-binding. Secondary, as long-acting mechanism, XSN was used to treat arrhythmia caused by coronary heart disease and viral myocarditis. Protection of myocardium, such as protection of I/R injury, antithrombosis and anti-apoptotic effect should be the essential effects of XSN. Intracellular sodium accumulation is a key pathophysiological mechanism in myocardial ischaemia/reperfusion (I/R) injury. Furthermore, it is thought that, Na_v_1.5, alongside NHE and other transporters, contributes to this sodium overload that can be reduced with application of lidocaine, suggesting that inhibition of I_Na_ may attenuate I/R injury. Previous work has shown XSN is cardioprotective in I/R injury induced in isolated rat hearts. Given the effect of ISL in inhibiting Na_v_1.5, it is possible that LSN and ISL reported in the present study are responsible for some of XSN’s cardioprotective actions in this way.

Besides of calcium overload, another reason of myocardium injury was caused by apoptosis of cardiomyocyte. The p53 tumor suppressor is one of the major apoptosis signaling pathways. It regulates a wide variety of genes involved in apoptosis, growth arrest, or senescence in response to genotoxic or cellular stress. The p53 can promote apoptosis through interactions with Bcl-2 family proteins in the cytoplasm. In addition, HIF1 alpha was also downregulated by the top1 component berberine (Mao et al., 2018). Berberine inhibits doxorubicin-induced cardiomyocyte apoptosis (Lv et al., 2012).

Inflammatory response is well recognized as a critical contributor for the development and complications of atherosclerosis cardiovascular disease (ASCVD), including myocardial infarction (MI), heart failure, and stroke, which involve complex interactions between multiple biological processes.

Besides of cardiovascular pathways, several diabetic pathways were also obtained. In TCM system, disease with diabetes-related symptoms is called “*Xiaoke*,” which is primarily caused by deficiency of Yin with dryness and heat syndromes as the secondary cause (Tong et al., 2012). As both PHHD and *Xiaoke* partially shared the pathogenesis of heat, it is understandable to share some biological pathways in common. In type II diabetes mellitus, insulin secretion, integration of energy metabolism pathways, KCNJ11, and ABCC8 were the common targets, which may be regulated by XSN. The beta‐cell ATP‐sensitive potassium (KATP) channel is a key component of stimulus‐secretion coupling in the pancreatic beta‐cell. The channel coupled metabolism to membrane electrical events bringing about insulin secretion. The channel consists of four subunits of the inwardly rectifying potassium channel Kir6.2 and four subunits of the sulfonylurea receptor 1 (SUR1).

Beyond of these targets, several targets including fatty acid synthase (FASN), pyruvate kinase L/R (PKLR), acetyl-CoA carboxylase (ACC, alpha unit encoded by ACACA), transketolase (TKT), free fatty acid receptor 1(FFAR1), glucagon-like peptide 1 receptor (GLP1R), and so on were also regulated by XSN. These targets are involved in the bioprocess of glycolysis, fatty acid synthesis, and pentose phosphate pathway, which are key steps of glucose and lipid metabolism. Through these mechanisms, XSN may improve fatty acid storage and convert it into glycogen. Lipid metabolism disorder has been reported being related to the TCM heat syndrome (Lv et al., 2012), which also suggest XSN regulate glucose and lipid metabolism to act as removing the extra heat based on the TCM theories.

In summary, the mechanism of XSN on treating PHHD arrhythmia may act as follows: on quick-acting aspect, XSN balanced the ion current of the heart by regulating multiple ion channels to terminate cardiac arrhythmia; on the long-acting aspect, XSN protects the heart from I/R injury, inhibits the apoptosis of cardiomyocyte, and improves glucose and lipid metabolism. Part of the mechanism of XSN on treating PHHD syndrome by sharing pathways with insulin secretion or glucose and lipid metabolism also suggest the potential therapeutic effect of diabetes and diabetic cardiopathy.

## Data Availability Statement

All datasets generated for this study are included in the manuscript/**Supplementary Files**.

## Author Contributions

Y-LM and TW conceived and designed the study; TW and HS performed the electrophysiological experiments; XW discussed, analyzed and standardized the TCM symptoms; UP and ML analyzed and discussed the biological functions of the pathways; CC provided experimental equipment and technical support for cell culturing; TW and Y-LM wrote the paper.

## Funding

This work was supported by grants from the Chinese Medicine Research Fund, University of Oxford. The grant was funded by Shaanxi Momentum Pharmaceutical Co.,Ltd.

## Conflict of Interest

The authors declare that this study received funding from Shaanxi Momentum Pharmaceutical Co., Ltd. The funder was not involved in the study design, collection, analysis, interpretation of data, the writing of this article or the decision to submit it for publication.

## Supplementary Material

Table S1 XSN component library.

Table S2 XSN component target relationships.

Table S3 XSN Target Weight Coefficient type.

Table S4 PHHD-arrhythmia target spectrum.

Table S5 XSN PHHD Pathway.
